# Lemierre’s Syndrome Due to Methicillin-Resistant *Staphylococcus aureus*

**DOI:** 10.1177/2324709619890967

**Published:** 2019-12-03

**Authors:** Rolando A. Zamora Gonzalez, Juan C. Sarria, Nicole A. Christians, Michelle Baliss

**Affiliations:** 1 University of Miami/Jackson Memorial Hospital, Miami, FL, USA; 2 University of Texas Medical Branch, Galveston, TX, USA

**Keywords:** Lemierre’s syndrome, facial vein thrombosis, septic emboli, methicillin-resistant *Staphylococcus aureus*

## Abstract

Lemierre’s syndrome is an uncommon and potentially fatal complication of oropharyngeal and facial infections. It involves an associated septic thrombophlebitis, bacteremia, and septic emboli. Traditionally, compromise of the internal jugular vein has been described in conjunction with an infection caused by anaerobes, especially, *Fusobacterium necrophorum*. In recent years, however, variant forms have been appearing, including other vessel compromise and other etiologic agents. We present the case of Lemierre’s syndrome in a 31-year-old male with facial vein thrombosis, septic emboli to the lungs, and bacteremia caused by methicillin-resistant *Staphylococcus aureus.* We hope that this case will raise awareness about variant presentations and promote a timely diagnosis and appropriate treatment of this potentially fatal infection.

## Introduction

In 1936, André Lemierre published the original description of what later became known as Lemierre’s syndrome.^1^ He described a septicemia produced by an anaerobic infection followed by local thrombophlebitis and septic emboli. Subsequently, with the advent of antimicrobials, the prevalence of this syndrome significantly decreased and became a “forgotten disease.”^2^ More recently, however, there appears to be a resurgence with atypical presentations and etiologies.^3^ In this article, we report a case of Lemierre’s syndrome caused by methicillin-resistant *Staphylococcus aureus* (MRSA) and discuss the changing aspect of this fascinating infection.

## Case

**Figure 1.**
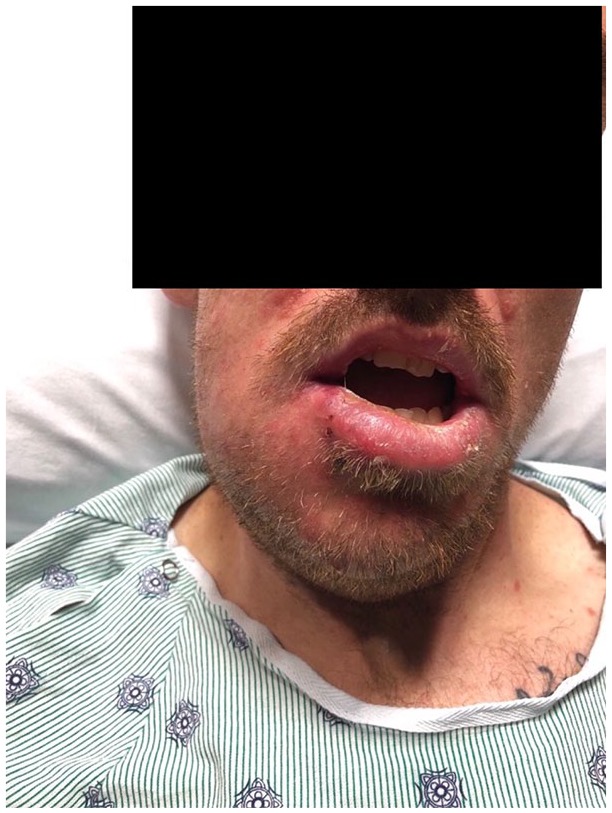
Right submandibular edema on initial presentation.

**Figure 2.**
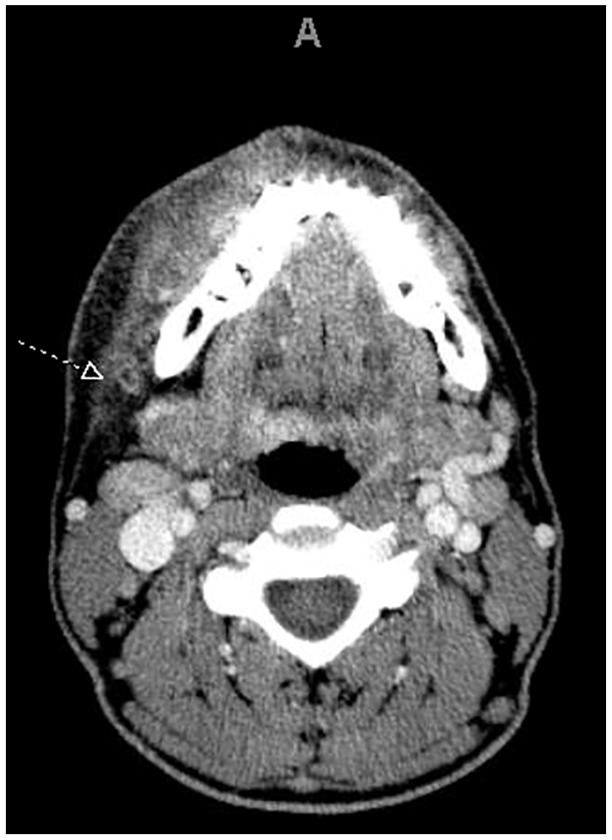
Contrast-enhanced computed tomography scan of neck showing right facial vein thrombosis (arrow) with surrounding inflammation.

**Figure 3.**
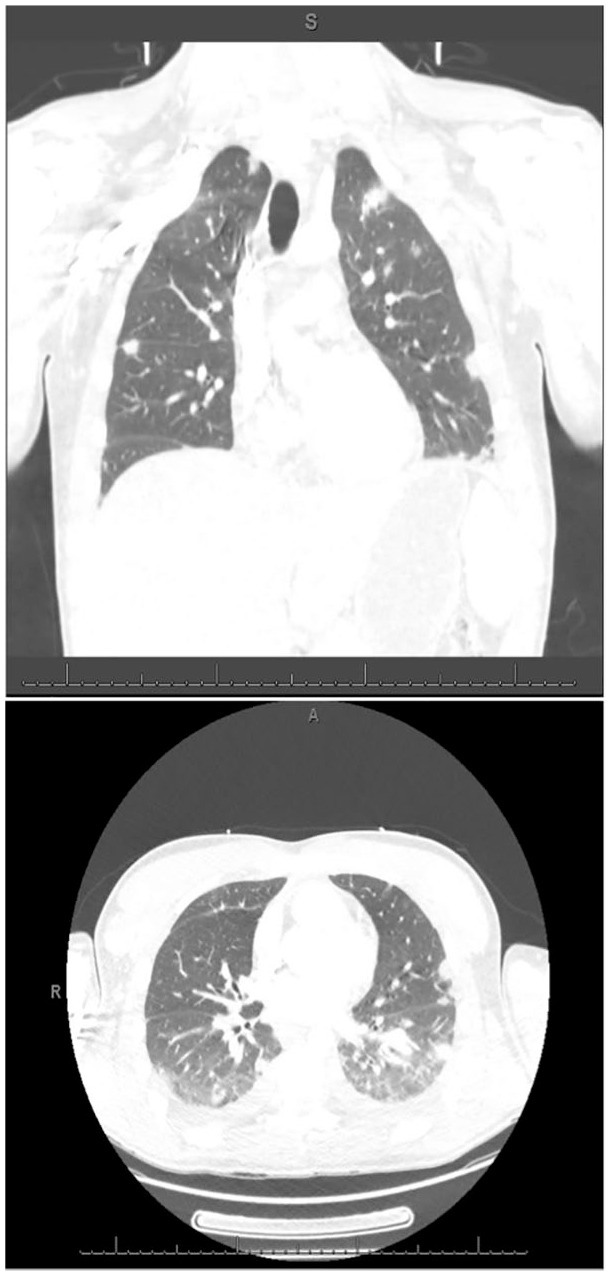
Computed tomography scan of the chest showing bilateral pulmonary nodules suggestive of septic emboli.

A 31-year-old male was admitted to the hospital with a 4-day history of fever, diaphoresis, and right submandibular edema. He also complained of sore throat, dyspnea, and pleuritic chest pain. He had a past history of HIV infection since 2017 and genital chlamydia infection. He was not taking antiretrovirals due to lack of compliance with his regular follow-up visits and medications. He endorsed alcohol, tobacco, and marijuana use but denied other illicit or intravenous drug use. On physical examination, he appeared acutely ill. He was febrile (39.4°C), tachycardic (143 heart rate), normotensive (121/66 mm Hg), and tachypneic (28 respiratory rate). Oxygen saturation was 99% on room air. He had right submandibular edema and a small orolabial ulcer with mild purulent drainage (Figure 1). Bilateral tonsillar hypertrophy without exudates was also noted. There were no signs of dental or periodontal infection. Pulmonary examination revealed decreased breath sounds in the left lower lung field and bibasilar crackles. The rest of his examination was unremarkable. Laboratory studies revealed a white blood cell count of 26 200 cells/µL (85% neutrophils, 8% bands). Arterial blood gases and routine chemistries were normal. CD4 count was 356 cells/µL. Chest X-ray showed bilateral nodular opacities and a small left pleural effusion. Computed tomography (CT) scan of the neck showed right-sided submandibular inflammation, facial vein thrombosis, and reactive cervical lymphadenopathy (Figure 2). CT scan of the chest revealed numerous subpleural and intraparenchymal pulmonary nodules, some with spiculated cavitation (Figure 3). Small bilateral pleural effusions, a large consolidative opacification in the left lower lobe, and bibasilar ground glass opacities were also noted. CT angiogram showed partial filling defects suggestive of right pulmonary artery and left anterior segment pulmonary embolism.

The patient was started on intravenous vancomycin and piperacillin/tazobactam. Piperacillin/tazobactam was discontinued after isolation of MRSA from blood cultures with no gram-negative or anaerobic growth. MRSA was also isolated from a drainage culture of the superficial orofacial ulcer. A transthoracic echocardiogram showed no valvular lesions or signs suggestive of infectious endocarditis. He initially received anticoagulation with intravenous heparin for 5 days and then oral apixaban for 5 additional days, but it was later discontinued.

**Figure 4.**
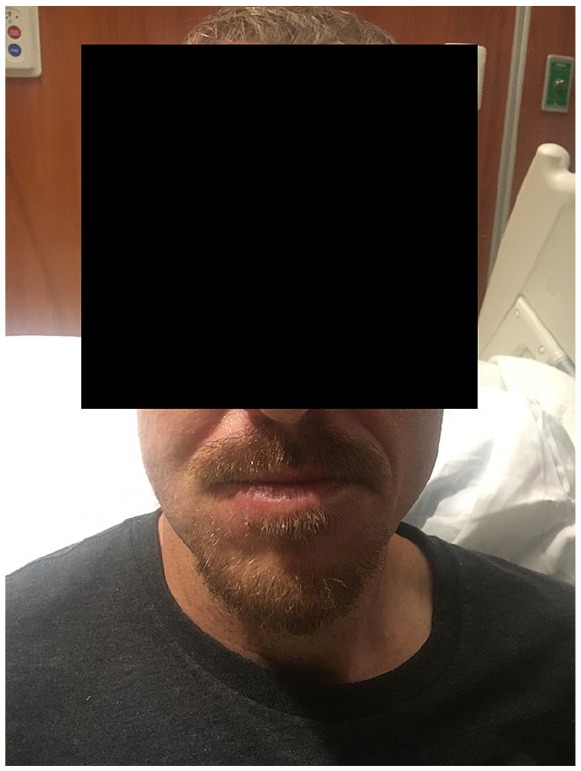
Patient after 10 days of treatment.

The patient responded well to treatment, and after 1 week, his submandibular edema subsided (Figure 4). He was discharged from the hospital after receiving 2 weeks of intravenous vancomycin and completed a 4-week course as an outpatient. This was administered via a peripherally inserted central catheter at a skilled nursing facility with monitoring of routine laboratories and vancomycin levels. At a 2-month follow-up outpatient appointment, he was doing well. He was instructed to keep regular follow-up visits for his HIV care.

## Discussion

Our case had features consistent with Lemierre’s syndrome including right submandibular inflammation, facial vein thrombosis, bacteremia, and metastatic pulmonary emboli. This syndrome has traditionally been described as an internal jugular vein septic thrombophlebitis, septicemia, and distant septic emboli (usually involving the lungs) in association with an oropharyngeal infection.^4^ Variations to this presentation, however, can occur. In his original description,^1^ Lemierre himself mentioned that the primary infection might arise from other sites, including inflammatory lesions of the mouth and jaw, as it was seen in our case. In addition, the internal jugular may not be the primary vein involved. Facial vein thrombosis has been described especially in infections of oropharyngeal or facial origin, as was also seen in our case. To our knowledge, only 6 cases of Lemierre’s syndrome presenting with facial vein involvement have been reported (Table 1).

**Table 1.** Seven Cases of Lemierre’s Syndrome Presenting With Facial Vein Thrombophlebitis.

Reference	Year Published	Vein(s) Involved
Risoud et al^4^	2016	Left facial, left anterior jugular
Nguyen-Dinh et al^9^	2002	Internal jugular, facial, anterior jugular
Kisser et al^10^	2012	Right facial
Iizuka et al^11^	2013	Left facial, junction with internal jugular
Karnov et al^12^	2014	Left facial
Cuddy et al^13^	2018	Left facial
Present report	2019	Right facial

The etiologic agent isolated in our case was atypical. Lemierre originally described several species of anaerobes that live as saprophytes in various cavities of the body, including the mouth and pharynx, as the primary pathogens implicated, and in particular, *Fusobacterium necrophorum*.^1^ Other pathogens including non-anaerobic species are implicated less frequently. A review of the literature published in 2002 by Chirinos et al^5^ showed that *Fusobacterium necrophorum* was present in 81.7% of 109 cases analyzed. Other pathogens found included *Bacteroides* sp, *Streptococcus* sp, *Staphylococcus epidermidis, Enterococcus* sp, and *Proteus* sp. *Staphylococcus aureus* was not reported in this series. Another literature review by Chanin et al^6^ published in 2011 identified 11 cases implicating *S aureus*; all reported since 2002. We conducted a MEDLINE search using the terms “Lemierre’s syndrome” and “*Staphylococcus aureus*” and identified 15 additional cases, all reported since 2010. These data suggest that *S aureus* may have become an important cause of Lemierre’s syndrome in recent years. It is unclear if decreased frequency of traditional more susceptible pathogens or an emergence of *S. aureus* has played a role. Definite conclusions cannot be drawn based on the limited data available. However, it is worth noting that this organism is known to promote coagulation by secreting 2 coagulases, staphylocoagulase and von Willebrand factor binding protein that activate prothrombin to generate fibrin. This coagulase activity is essential for the formation of *S. aureus*–fibrin-platelet microaggregates and for the homing of the organism to the vascular wall under flow. These mechanisms contribute to immune evasion and disease pathogenesis.^7^

Molloy et al stated that despite often being described as a condition of the pre-antibiotic era, it now seems that Lemierre’s syndrome belongs to our modern era of increasingly virulent and antibiotic-resistant organisms.^8^ Sherer and Mishal stated in their editorial “The Changing Face of Lemierre’s Syndrome”^3^ that in this post-antimicrobial era, some of the classical characteristics of the disease have changed. They also concluded after analyzing several cases that one should expect “variations or incomplete forms” of the disease due to the widespread use of antibiotics. Chirinos et al^5^ similarly stated that the typical course of this syndrome has changed since Lemierre’s original description, most likely as a consequence of widespread antibiotic usage in pharyngeal infections.

We believe that increased awareness about atypical presentations and adding empiric antimicrobial coverage against MRSA should be considered whenever there is suspicion of Lemierre’s syndrome.

## Conclusion

Our case illustrates an atypical presentation of Lemierre’s syndrome due to MRSA. Clinicians should be aware of the changing clinical features of this syndrome as earlier recognition and pathogen-specific antimicrobial treatment will lead to improved outcomes.

## References

[1] Lemierre A. On certain septicaemias due to anaerobic organisms. Lancet. 1936;1:701-703.

[2] Karkos PD Asrani S Karkos CD, et al Lemierre’s syndrome: a systematic review. Laryngoscope. 2009;119:1552-1559. 19554637 10.1002/lary.20542

[3] Sherer Y Mishal J. The changing face of Lemierre’s syndrome. Isr Med Assoc J. 2003;5:819-820. 14650111

[4] Risoud M Mortuaire G Chevalier D Rysman B. Atypical Lemierre syndrome. Eur Ann Otorhinolaryngol Head Neck Dis. 2016;133:123-124. 26718846 10.1016/j.anorl.2015.12.001

[5] Chirinos JA Lichststein DM Garcia J Tamariz LJ. The evolution of Lemierre Syndrome report of 2 cases and review of the literature. Medicine (Baltimore). 2002;81:458-465. 12441902 10.1097/00005792-200211000-00006

[6] Chanin JM Marcos LA Thompson BM, et al Methicillin-resistant *Staphylococcus aureus* USA300 clone as a cause of Lemierre’s syndrome. J Clin Microbiol. 2011;49:2063-2066. 21430106 10.1128/JCM.02507-10 PMC3122675

[7] Peetermans M Verhamme P Vanassche T. Coagulase activity by *Staphylococcus aureus*: a potential target for therapy? Semin Thromb Hemost. 2015;41:433-444. 25973589 10.1055/s-0035-1549849

[8] Molloy A Towersey G Shackleton D The changing face of an old disease: case report of nonclassical Lemierre’s syndrome caused by a Panton-Valentine leucocidin-positive methicillin-susceptible *Staphylococcus aureus* isolate. J Clin Microbiol. 2012;50:3144-3145. 22760040 10.1128/JCM.00939-12 PMC3421801

[9] Nguyen-Dinh K Marsot-Dupuch K Portier F, et al Lemierre syndrome: usefulness of CT in detection of extensive occult thrombophlebitis. J Neuroradiol. 2002;29:132-135. 12297736

[10] Kisser U Gurkov R Flatz W, et al Lemierre syndrome: a case report. Am J Otolaryngol. 2012;33:159-162. 21345516 10.1016/j.amjoto.2010.12.008

[11] Iizuka T Nagaya K Sasaki D, et al Atypical Lemierre syndrome, thrombophlebitis of the facial vein. Am J Emerg Med. 2013;31:460.e1-460.e3. 10.1016/j.ajem.2012.08.006 23041486

[12] Karnov KKS Lilja-Fischer J Randrup TS Isolated facial vein thrombophlebitis: a variant of Lemierre syndrome. Open Forum Infect Dis. 2014;1:ofu053. 25734123 10.1093/ofid/ofu053 PMC4281778

[13] Cuddy K Saadat N Khatib B Patel A. Necrotizing lip infection causing septic thrombophlebitis of the neck: a rare variant of Lemierre syndrome. J Oral Maxillofac Surg. 2018;76:134-139. 28651067 10.1016/j.joms.2017.05.030

